# The role of perfusion and diffusion MRI in the assessment of patients affected by probable idiopathic normal pressure hydrocephalus. A cohort-prospective preliminary study

**DOI:** 10.1186/s12987-017-0072-3

**Published:** 2017-09-12

**Authors:** Francesco Tuniz, Maria Caterina Vescovi, Daniele Bagatto, Daniela Drigo, Maria Cristina De Colle, Marta Maieron, Miran Skrap

**Affiliations:** 1 Department of Neurosurgery, AOU-UD “Santa Maria della Misericordia”, Piazzale S.M. della Misericordia, 33100 Udine, Italy; 2 Department of Neuroradiology, AOU-UD “Santa Maria della Misericordia”, Udine, Italy; 3 Institute of Epidemiology, AOU-UD “Santa Maria della Misericordia”, Udine, Italy; 4 Department of Physics, AOU-UD “Santa Maria della Misericordia”, Udine, Italy

**Keywords:** Diffusion, Hydrocephalus, iNPH, MRI, Perfusion, Shunt

## Abstract

**Background:**

Invasive tests measuring resistance to cerebral spinal fluid (CSF) outflow and the effect of temporary drainage of CSF are used to select candidates affected by idiopathic normal pressure hydrocephalus (iNPH) for shunt surgery. Neither test, however, completely excludes patients from treatment. Perfusion and diffusion magnetic resonance imaging (MRI) are non-invasive techniques that might be of value in selecting patients for surgical treatment and understanding brain changes in iNPH patients. The aim of this study was to understand the role of perfusion and diffusion MRI in selecting candidates for shunt surgery and to investigate the relationship between cerebral perfusion and possible microstructural changes in brain tissue before and after invasive tests, and after ventricular-peritoneal (VP) shunt implantation, to better clarify pathophysiological mechanisms underlying iNPH.

**Methods:**

Twenty-three consecutive patients with probable iNPH were included in this study. Patients underwent a clinical and neuroradiological evaluation before and after invasive tests, and after surgery. Only patients who showed a positive result in at least one of the invasive tests were submitted for VP shunt implantation. Perfusion and diffusion magnetic resonance imaging (MRI) was performed before and after invasive tests and after shunt surgery.

**Results:**

Thirteen patients underwent surgery and all showed clinical improvement after VP shunt implantation and a significant increase in perfusion in both periventricular white matter (PVWM) and basal ganglia (BG) regions. The 10 patients that did not have surgery showed after invasive tests, a significant reduction in perfusion in both PVWM and BG regions. Comparing the changes in perfusion with those of diffusion in positive patients we found a significant positive correlation in BG and a significant inverse correlation in PVWM area.

**Conclusions:**

Perfusion MRI is a non-invasive technique that could be useful together with invasive tests in selecting patients for surgical treatment. Furthermore, the relationship between perfusion and diffusion data could better clarify pathophysiological mechanisms underlying iNPH. In PVWM area we suggest that interstitial edema could reduce microvascular blood flow and interfere with the blood supply to these regions. In BG regions we suggest that a chronic hypoxic insult caused by blood hypo-perfusion produces a chronic cytotoxic edema. Both in PVWM and in BG regions, pathophysiological mechanisms could be modified after VP-shunt implantation.

## Background

Idiopathic normal pressure hydrocephalus (iNPH) is a condition characterized by ventricular enlargement and normal intracranial pressure (ICP) caused by disturbed cerebral spinal fluid (CSF) dynamics [1]. The cause is still unknown. The iNPH signs are typically subcortical, characterized by slow progressive impairment of gait and balance, cognitive deterioration and urinary incontinence [2]. Treatment of iNPH patients with ventricular-peritoneal or ventricular-atrial shunts is successful, with an improvement rate of more than 80% in recent short-term studies, and an acceptable complication rate [3–7]. At present, clinicians have two invasive predictive tests to select patient candidates for surgery: a test measuring compliance of craniospinal space or resistance to CSF outflow (Rout), and a test measuring the effect on symptoms of temporary drainage of CSF (CSF tap test) [8–10]. These tests are, however, not totally specific or sensitive and can be used for selecting patients for shunt surgery but not for excluding patients from treatment [11, 12]. Better methods for the identification of responders and non-responders are required. Cerebral blood flow (CBF) is reduced in iNPH patients, mainly in the frontal cortex and in accordance with the subcortical symptomatology, in the basal ganglia, in the thalami and also in the periventricular white matter (PVWM) [13–19]. As the subcortical and periventricular regions seem to be of special interest in iNPH, magnetic resonance (MR) perfusion imaging with its relatively high resolution and sensitivity for deep structures might be of value as a diagnostic and predictive tool [20]. Some authors have also investigated the role of diffusion MRI in determining brain parenchymal damage in PVWM and basal ganglia (BG) areas and the role of apparent diffusion coefficient (ADC) in predicting surgical outcome [21–26].

The aims of this study were to investigate (a) the relationship between cerebral perfusion and microstructural damage of brain tissue as measured by perfusion and diffusion MRI in PVWM and BG areas before, after invasive tests, and after surgery and (b) the potential role of perfusion and diffusion MRI in selecting patients for VP-shunt implantation.

## Methods

### Patients selection and clinical evaluation

Twenty-three patients diagnosed with probable iNPH were prospectively included between January 2013 and December 2014. Inclusion criteria for probable iNPH were based on iNPH Guidelines: presence of a gait disturbance in combination with cognitive and/or urinary symptoms and an Evans’ Index > 0.30 in the absence of any known cause for secondary hydrocephalus [2]. All patients underwent neuropsychological, physiotherapeutic and neurological examinations, and performance was assessed in the four domains of gait, neuropsychology, balance and continence, yielding separate scores as well as a total score on a recently published iNPH scale [27]. Domain and total scores all range from 0 to 100 with 0 representing the most severe condition and 100 representing normal performance among healthy individuals aged 70–74. This study was approved by our Institutional Review Board and an informed consent was acquired from each patient involved in this study.

### Invasive tests

A lumbar infusion test and a tap test were performed on each patient in same procedure. Lumbar puncture was performed in the morning and mean CSF basal pressure was recorded. A lumbar infusion test was then performed using the constant rate infusion method and Rout was calculated. After the infusion test was completed, CSF was drained until the pressure returned to baseline. Then, a tap test was performed, draining 50 ml of CSF. A value of Rout greater than 13 mmHg/min/ml was considered a predictor for good outcome after shunt implantation [28, 29], as well as a good response to the CSF tap test. The response to CSF tap test was expressed as the mean of the percentage change in all motor and psychometric tests compared with the previously achieved results. An increase of 5% was considered significant [11].

### Patient groups

Patients were divided into two groups. Those who resulted positive in at least one of the invasive tests underwent ventricular-peritoneal shunt implantation (positive patients, PP); when both invasive tests were negative, patients were denied the surgical procedure (negative patients, NP).

### Surgical procedure

All positive patients underwent ventricular-peritoneal shunt implantation. The right ventricular frontal horn was chosen for all patients and a Codman^®^ Hakim^®^ programmable valve with pre-chamber was implanted. The pressure of the valve was set with regards to the level of clinical impairment, the neuroradiological images and response to the invasive tests. Two days after surgical intervention a head CT-scan was performed to rule out surgical complications and to verify the correct placement of the ventricular catheter. All patients were discharged from hospital 5 to 7 days after ventricular-peritoneal shunt implantation with a good clinical recovery. One month after the ventricular peritoneal shunt positioning, positive patients repeated the clinical evaluation.

### MRI study

MRI was performed for each patient before and after invasive tests and one month after ventricular peritoneal shunt implantation (timing was chosen to permit good recovery after the surgical procedure). For each patient, perfusion and diffusion sequences were acquired and values for relative cerebral blood flow (rCBF) and apparent diffusion coefficient (ADC) were extrapolated.

Examinations were performed on a 3.0T magnet (Achieva; Philips Medical Systems, Best, The Netherlands).

At the first MRI examination, all patients before diffusion tensor imaging (DTI) and perfusion-weighted imaging (PWI) studies, also underwent a standard brain MRI protocol that included non-enhanced axial inversion recovery (IR) T1-weighted images, sagittal 3D T2-weighted images and axial fluid attenuated inversion recovery (FLAIR) sequences. During the second and third MRI examinations, only DTI and PWI sequences were performed.

### Perfusion MRI

Dynamic Susceptibility Contrast-Enhanced (DSC) MR imaging was performed using gradient echo planar T2*−weighted sequence with the following parameters: TR = 1708 ms, TE = 40 ms, inplane resolution = 1.75 × 1.75 mm, slice thickness = 4 mm, number of slices = 25, slice GAP = 0 mm.

**Fig. 1:**
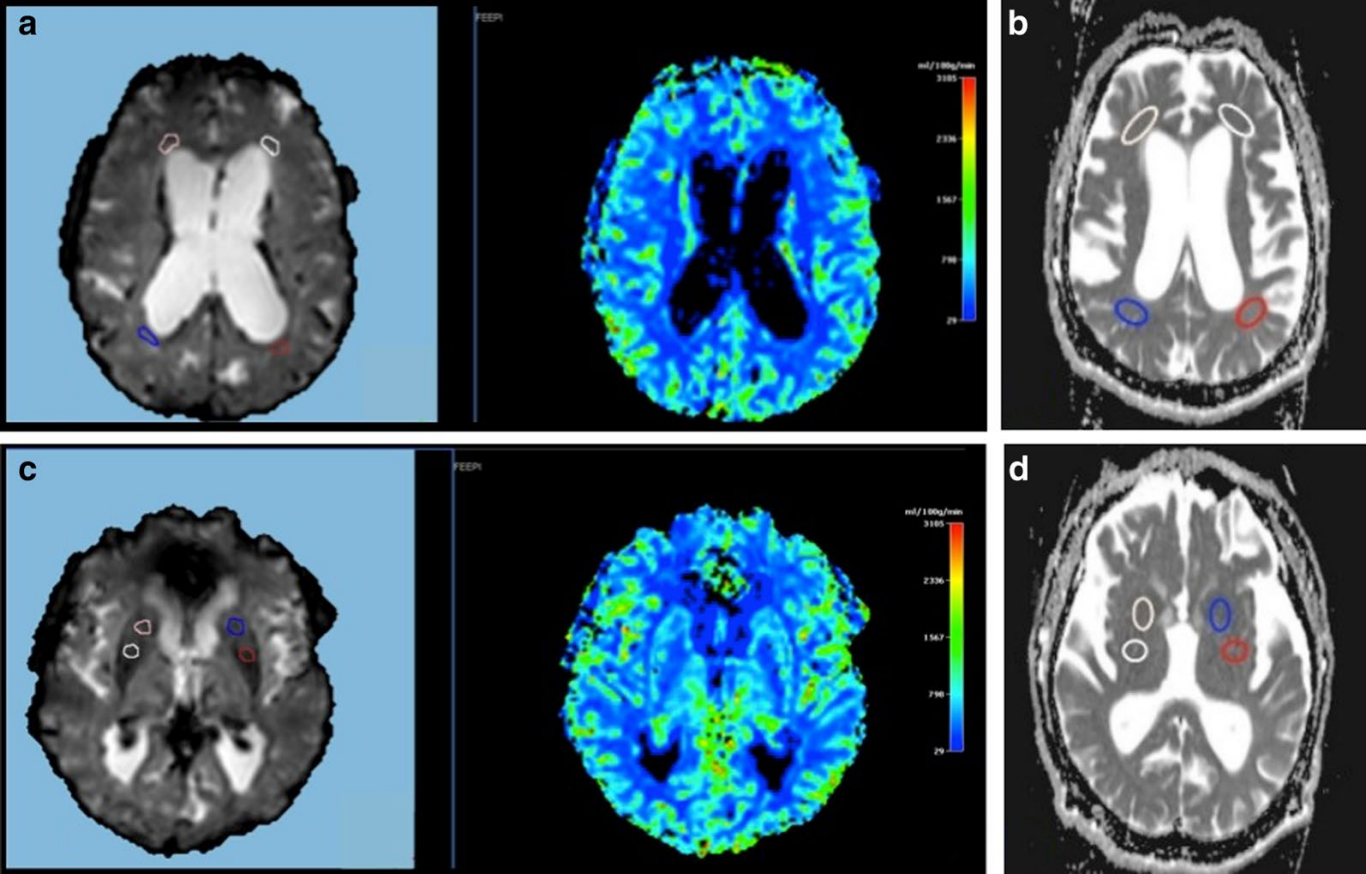
MR images of patient G. **a**, **c** Regions of interest (ROIs) positioned on perfusion maps in periventricular and basal ganglia region respectively. **b**, **d** ROIs positioned on ADC diffusion maps in periventricular and basal ganglia region respectively

Ten seconds after the start of image acquisition, a bolus of a 1.0 mmol/ml gadobutrol formula (Gadovist; Schering Bayer Pharma, Leverkusen, Germany) in a dose of 0.1 mmol/kg of body weight (as indicated by the manufacturer) was injected via a 20-gauge catheter placed in the antecubital vein. Contrast administration was performed using an automatic injector (MEDRAD Spectris Solaris EP MR injection System, Indianola PA, USA) at a rate of 5 ml/s and was followed by a saline bolus (20 ml at 5 ml/s). The dynamic images were post-processed using the vendor specific software on the MRI scanner. For each patient, two different examiners, one neurosurgeon and one neuroradiologist, placed sixteen regions of interest (ROIs) in the same basal ganglia region (i.e. nucleus caudatus, putamen, striatum) and in PVWM area. Six ROIs were also positioned in cortical occipital region. Careful positioning avoided inclusion of other anatomical structures. During image post-processing the two examiners were blinded to patient clinical evaluations and invasive test results, thus ensuring that results were unbiased. As an example, Fig. 1 show ROIs positioned in the periventricular and basal ganglia regions. For each ROI value the relative cerebral blood flow (rCBF), relative cerebral blood volume (rCMV) and mean transit time (MTT) were calculated. In the present study, we used the rCBF as parameter of choice, using the mean cortical occipital rCBF as internal reference.

### Diffusion study

Diffusion-weighted images were acquired before administration of contrast medium using a diffusion-weighted single-shot echo-planar sequence with diffusion gradients along the fifteen directions and effective b-values of 0 and 1000 s/mm^2^. ADC and fractional anisotropy (FA) maps were automatically generated by the software employed in the MRI scanner console. Scan parameters of DTI sequence were as follows: TR = 7204 ms, TE = 62 ms, inplane resolution = 1.62 × 1.62 mm, slice thickness = 2 mm, number of slices = 60, slice GAP = 0 mm.

ADC values were calculated in ROIs positioned in basal ganglia and PVWM area and overlapped the perfusion ROIs.

### Statistical analysis

Descriptive analysis was performed: median, interquartile range (IQR), mean and standard deviation (SD) were calculated. The parameters of perfusion and diffusion between PP versus NP groups and within subjects were compared by Wilcoxon Mann–Whitney test and Wilcoxon signed rank sum test, respectively. For statistical analysis we applied relative perfusion values, which were calculated using estimates for the occipital cortex as an internal reference [20]. Differences in mean values of perfusion and diffusion from baseline to post-surgery were estimated for each patient. Spearman correlation was calculated. The alpha level was set to 0.05 for all tests. The statistical analysis was performed using Epi Info™ v. 3.5.1 and Microsoft Excel software.

## Results

Thirteen patients (56.53%) had at least one positive result in the invasive tests. We referred to this patient group as positive patients (PP). Ten patients (43.47%) conversely had no positive result either in the lumbar infusion test or in the tap test. We referred to this patient group as negative patients (NP).

In the PP group, six patients were male (46.15%) and seven patients were female (53.85%); in the NP group, four patients were male (40%), and six patients were female (60%). For the PP the mean age was 72.5 ± 6.94 years, whereas for NP mean age was 70.7 ± 6.78 years. Regarding invasive tests results in the PP group 10 patients had a Rout greater than 13 mmHg/min/ml (76.92%) and 10 patients had a positive result in the tap test, showing a clinical improvement greater than 5% (Table 1). In the NP group no patient showed clinical improvement after the tap test or a Rout greater than 13 mmHg/min/ml (Table 2).

**Table 1:** Positive patients (PP) personal and clinical data

Patient	Sex	Age (years)	Rout (mmHg/min/ml)	Tap test	Total clinical score and its % variation				
					Basal	Post-test	% Variation	Post-surgery	% Variation
A	Male	82	10.40	Positive	60.75	78.25	28.81	84.75	39.51
B	Female	72	13.20	Positive	54.25	69.50	28.11	88.25	62.67
C	Female	68	17.90	Positive	36.50	79.75	25.59	88.25	38.98
D	Female	69	6.10	Positive	55.00	76.50	39.09	95.75	74.09
E	Female	73	22.50	Positive	65.75	81.00	23.19	89.75	36.50
F	Female	74	14.90	Negative	62.75	62.75	0.00	82.25	31.08
G	Male	77	18.20	Positive	53.25	84.75	59.15	88.25	65.73
H	Male	56	15.00	Positive	84.75	98.75	16.52	98.75	16.52
I	Female	75	13.60	Positive	57.75	82.25	42.42	82.25	42.42
L	Male	80	14.00	Positive	44.00	69.50	57.95	69.50	57.95
M	Male	65	11.30	Positive	74.50	88.50	18.79	93.25	25.17
N	Male	79	15.00	Negative	59.25	61.75	4.22	79.75	34.60
O	Female	73	13.60	Negative	64.50	64.50	0.00	84.75	31.40

**Table 2:** Negative patients (NP) personal and clinical data

Patient	Sex	Age (years)	Rout (mmHg/min/ml)	Tap test	Total basal clinical score
1	Female	75	8.60	Negative	49.00
2	Male	67	5.10	Negative	22.50
3	Female	74	11.10	Negative	73.25
4	Male	59	11.40	Negative	78.25
5	Female	66	10.00	Negative	69.50
6	Female	72	9.50	Negative	51.75
7	Female	69	7.90	Negative	45.75
8	Female	81	12.20	Negative	67.00
9	Male	79	10.40	Negative	66.00
10	Male	65	11.30	Negative	56.75

Considering clinical characteristics, in the PP group, clinical basal score ranged from 44.00 to 84.75 with an average value of 61.54 ± 10.13. In the NP group, baseline clinical score ranged from 22.50 to 78.25 with an average value of 57.97 ± 16.51. No significant differences were demonstrated between the two groups, either in total clinical score or singular domain.

After invasive tests were performed, 10 patients of the PP group showed clinical improvement with a clinical score increase of more than 5%. Average improvement rate was 26.45% ± 19.47% (Table 1). None of the NP showed clinical improvement greater than 5% after the tap test.

PP patients were clinically evaluated again 1 month after ventriculo-peritoneal shunt implantation. In the third clinical evaluation all PP patients showed clinical improvement after shunt implantation. Average improvement rate with respect to basal evaluation was 42.82% ± 17.15%. In Table 1 PP personal data, results of invasive tests and clinical score at basal, post-test and post-surgery evaluation are reported. In Table 2 the same data for NP are summarized.

Perfusion and diffusion values of PP group are reported in Table 3 for the periventricular and basal ganglia regions. Values were obtained during the baseline radiological study, after invasive test examination and after shunt implantation. In Table 4 the same values are summarized for the NP group.

**Table 3:** Positive patients (PP) perfusion and diffusion values

Patients	Perfusion mean values (rate)^a^						Diffusion mean values (ADC mm^2^/sec)^b^					
	Periventricular white matter			Basal ganglia			Periventricular white matter			Basal ganglia		
	Basal evaluation	Post-test evaluation	After surgery evaluation	Basal evaluation	Post-test evaluation	After surgery evaluation	Basal evaluation	Post-test evaluation	After surgery evaluation	Basal evaluation	Post-test evaluation	After surgery evaluation
A	0.85	1.11	1.34	1.46	2.22	2.45	1107.84	1075.96	1122.28	662.71	763.95	811.15
B	0.59	0.71	0.84	1.12	1.33	1.38	917.99	893.31	875.20	753.40	781.38	762.25
C	0.64	1.03	1.08	1.24	1.89	2.02	787.90	814.88	812.00	677.18	683.95	697.66
D	0.64	0.90	1.52	1.35	1.85	2.87	1271.25	1037.63	908.98	729.51	778.29	908.98
E	0.51	1.08	0.98	0.89	2.00	1.90	857.25	819.58	801.34	701.10	725.04	778.50
F	0.58	1.40	1.47	1.07	2.39	2.36	993.28	925.93	941.58	725.03	727.94	734.56
G	0.61	0.91	0.78	1.00	1.54	1.17	947.41	925.24	925.48	752.65	752.26	772.91
H	0.51	0.68	0.76	1.05	1.16	1.51	821.90	840.56	840.85	684.59	667.03	698.10
I	0.58	0.68	0.84	1.01	1.02	1.46	881.43	859.48	834.24	682.03	749.88	742.79
L	0.37	0.57	0.77	1.10	1.01	1.29	1116.04	1071.39	1056.63	675.35	621.00	746.25
M	0.91	1.02	1.24	1.42	1.78	2.12	872.51	832.69	841.56	694.99	672.75	734.56
N	0.59	0.71	0.84	1.12	1.33	1.38	917.99	893.31	875.20	753.40	781.38	762.25
O	0.64	0.90	1.52	1.35	1.85	2.87	1271.25	1037.63	908.98	729.51	778.29	908.98

^a^Perfusion rates are expressed relative to that in the occipital cortex

^b^Diffusion is expressed using apparent diffusion coefficient (ADC) parameter

**Table 4:** Negative patients (NP) perfusion and diffusion values

Patients	Perfusion mean values (rate)^a^				Diffusion mean values (ADC mm^2^/s)^b^			
	Periventricular white matter		Basal ganglia		Periventricular white matter		Basal ganglia	
	Basal evaluation	Post-test evaluation	Basal evaluation	Post-test evaluation	Basal evaluation	Post-test evaluation	Basal evaluation	Post-test evaluation
1	0.36	0.25	0.75	0.48	1638.29	1612.69	745.14	757.73
2	0.57	0.49	1.29	1.10	1351.23	1371.06	734.81	722.08
3	0.53	0.30	1.34	0.83	1067.76	1003.93	736.24	749.36
4	0.76	0.58	1.44	1.15	863.78	822.19	647.24	681.00
5	0.56	0.49	1.10	0.97	864.50	877.64	763.63	754.95
6	0.53	0.33	1.34	0.87	1156.39	1066.43	823.74	835.61
7	0.93	0.78	1.63	1.33	765.13	778.51	676.38	669.20
8	0.62	0.58	1.30	1.14	940.13	952.58	851.38	844.83
9	0.43	0.20	1.24	0.73	666.65	620.79	447.24	482.25
10	0.62	0.49	1.30	1.18	1454.26	1354.30	1000.11	1009.74

^a^Perfusion rates are expressed relative to that in the occipital cortex

^b^Diffusion is expressed using apparent diffusion coefficient (ADC) parameter

Basal values of perfusion and diffusion between the PP and NP groups were compared. No significant differences were demonstrated at basal evaluation either in the periventricular region, or in the basal ganglia region. The post-invasive test values of perfusion and ADC were also compared between the PP and NP groups and a significant difference was found for perfusion values in both the periventricular and the basal ganglia areas between two patient groups. No significant differences were demonstrated in either region for ADC values (Table 5).

**Table 5:** Median and IQR of perfusion and diffusion of PP and NP groups and statistical analysis

Region	RMN	Patient group	Time			p value**		
			Basal median value (IQR)	Post test median value (IQR)	Post surgery median value (IQR)	Basal vs. post-test	Basal vs. post-surgery	Post test vs. post-surgery
Periventricular region	Perfusion	PP	0.59 (0.58–0.64)	0.90 (0.71–1.03)	0.98 (0.84–1.34)	0.0014	0.0014	0.0156
		NP	0.56 (0.53–0.62)	0.49 (0.31–0.56)	–	0.0051		
		p value*	ns	0.003				
	Diffusion	PP	917.99 (872.51–1107.84)	893.31 (840.56–1037.63)	875.2 (840.85–925.48)	0.0071	0.0071	ns
		NP	1003.94 (863.96–1302.52)	978.25 (836.05–1282.33)	–	ns		
		p value*	ns	ns				
Basal ganglia	Perfusion	PP	1.12 (1.05–1.35)	1.78 (1.33–1.89)	1.9 (1.38–2.36)	0.0024	0.0015	0.0329
		NP	1.30 (1.25–1.25)	1.03 (0.84–1.15)	–	0.0051		
		p value*	ns	0.001				
	Diffusion	PP	701.10 (681.03–729.51)	749.88 (683.95–778.29)	762.25 (734.56–778.5)	ns	0.0015	0.0158
		NP	742.59 (690.98–808.71)	752.16 (691.27–816.14)	–	ns		
		p value*	ns	ns				

* Wilcoxon Mann–Whitney test

** Wilcoxon signed rank sum test

**Fig. 2:**
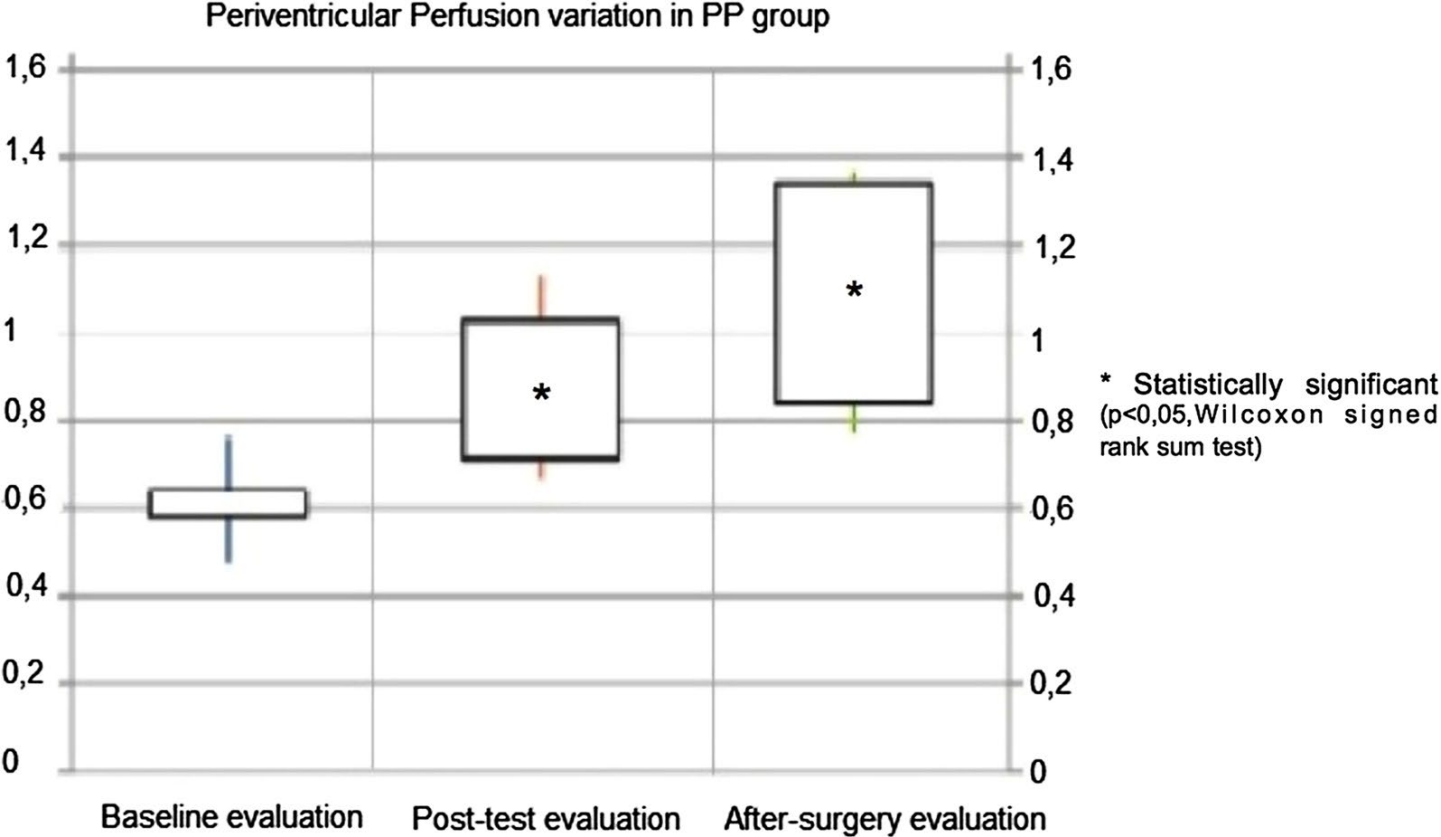
Periventricular white matter perfusion changes in positive patients (PP). On the vertical axis relative perfusion values are reported [mean rCBF Periventricular/mean rCBF Occipital]. Rectangles represent data inter-quartile range (IQR) and straight lines represent standard deviation (SD). Data are represented for baseline evaluation, after test evaluation and post-surgery assessment. Asterisk indicates when the variation was statistically significant

**Fig. 3:**
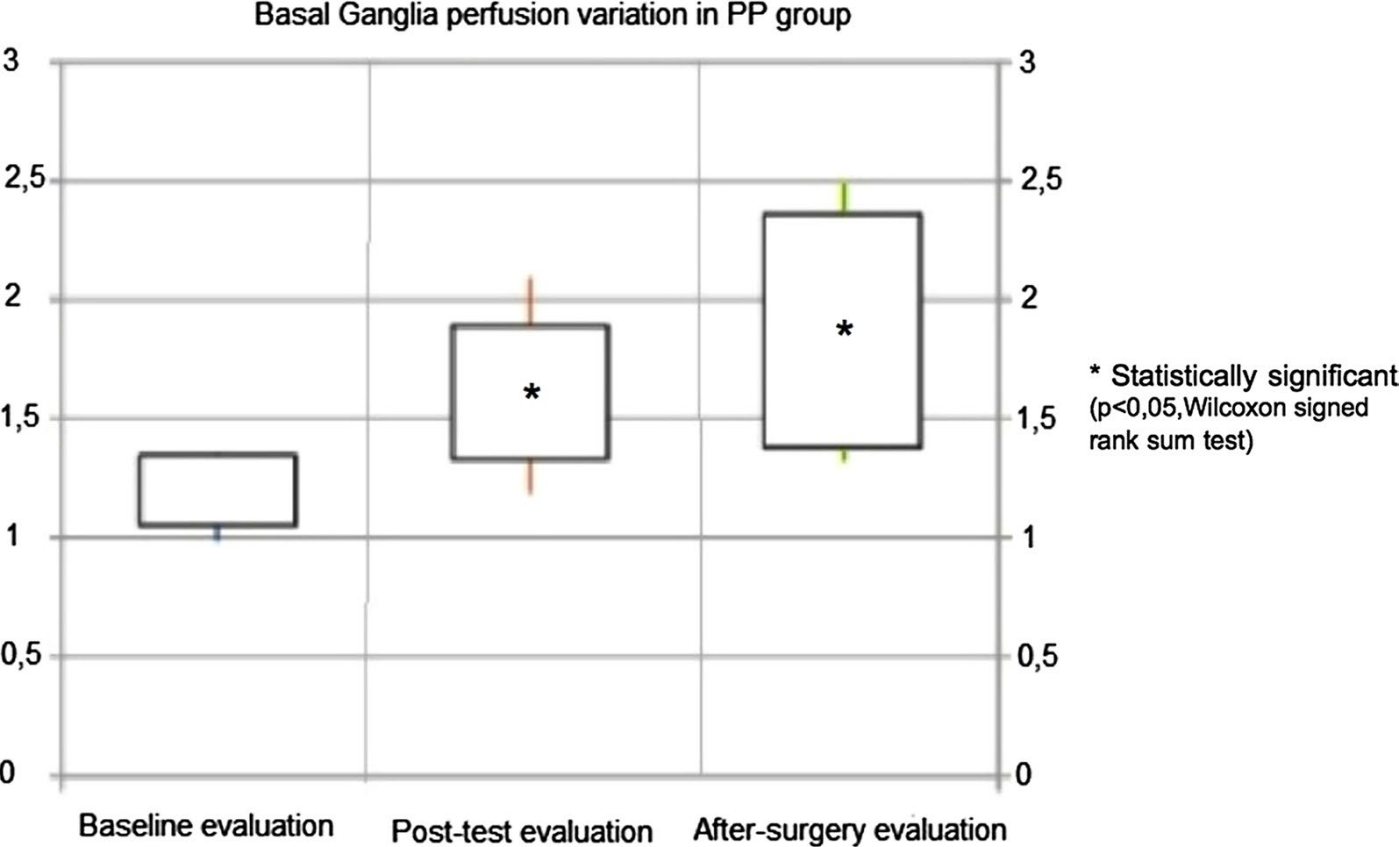
Basal ganglia perfusion changes in positive patients (PP). On the vertical axis relative perfusion values are reported [mean rCBF Basal Ganglia/mean rCBF Occipital]. Rectangles represent data inter-quartile range (IQR) and straight lines represent standard deviation (SD). Data are represented for baseline evaluation, after test evaluation and post-surgery assessment

Perfusion changes within the PP and NP groups were analysed from basal to post-test and after surgery procedure. A significant improvement of perfusion was found in PP group both in BG region and PVWM area, while NP group shows a significant decrease in the same regions. In Figs. 2 and 3 periventricular and basal ganglia perfusion variation from baseline evaluation to post-surgery assessment in positive patients (PP) are represented. When considering diffusion measurements within each group, results are different. We found a significant decrease of ADC values in PVWM area and a significant increase in BG regions in PP group. In NP group statistical significance was not reached either in PVWM area or in BG. In Table 5 the median and IQR of perfusion and diffusion of PP and NP groups and statistical analysis are reported.

We also investigated the relationship between the clinical score, ADC values, perfusion values and their variation in PP group. Only the correlations that have an impact on clinical and pathophysiological conditions are reported:

- Clinical score and periventricular ADC values in basal and post-test evaluation: a significant inverse correlation was demonstrated (r_s_ = −0.51 and r_s_ = −0.56 respectively). In post-surgery evaluation no significant correlation was detected (r_s_ = −0.46).
- Periventricular perfusion and ADC variation from basal to post-surgery evaluation: a significant inverse correlation was reached (r_s_ = −0.52).
- Spearman correlation of differential values of perfusion and ADC in basal ganglia from basal to post-surgery evaluation: a significant direct correlation was reached (r_s_ = 0.57).

## Discussion

Changes in cerebral blood perfusion in iNPH patients compared with aged-matched controls, and changes in brain tissue microstructure, have already been demonstrated by multiple studies [13, 14, 16–20, 24]. In this study we considered only patients with probable iNPH. Patients were recruited over a limited period of 24 months and were prospectively followed; clinical and radiological evaluations were made at the beginning, after invasive tests and after shunt procedure. The patient population was divided into two sub-groups: patients who had at least one positive response to invasive tests and who had undergone surgical treatment (PP group), and patients who did not show any improvement after the tap test or a significantly high Rout (NP group). After initial clinical data analysis, neither the total clinical score nor the singular domain score showed significant differences between the two groups of patients.

### Perfusion MRI

To evaluate cerebral perfusion we used a DSC MRI perfusion sequence, a non-invasive technique. This technique suffers from specific problems related to CBV and CBF quantification in absolute terms. For inter-subject comparison or follow-up evaluation relative perfusion estimates are usually used. We chose occipital cortical perfusion as the internal reference assuming that this region would be less affected by global perfusion reduction [19, 20, 30–32].

The PVWM and BG perfusion data obtained at the beginning of the study (basal values) are consistent with values available in the literature for iNPH [20]. In the baseline evaluation no significant differences were demonstrated in perfusion values between PP and NP groups either in PVWM region or in basal ganglia. Likewise no significant difference was obtained in ADC values. Given these results, the authors could not identify a radiological perfusion or diffusion marker for selecting patients to submit to surgery without carrying out invasive tests.

Perfusion values showed a different response between the two groups after performing the invasive tests. In the PP group, perfusion increased significantly both in the periventricular and basal ganglia areas and the same result was achieved after VP shunt. In the PP group we detected clinical improvement in the majority of the cases related to an increase of the CBF values after the invasive tests were performed. These results is are consistent with the ones in literature [32]. NP, on the other hand, showed a significant decrease in perfusion values after invasive tests both in PVWM and in basal ganglia areas and no clinical improvement was achieved in these patients. Significant differences were found for PVWM and basal ganglia perfusion values between PP and NP after performing the invasive tests.

Given the different perfusion patterns between PP and NP, we suggest that perfusion MRI could be useful together with invasive tests in selecting patients for surgical treatment. As an example, three PP patients (F) (N) (O) presented a Rout slightly above the threshold value and no clinical improvement after tap test. All these patients, however, showed a good improvement in perfusion values in PVWM and BG. Patients were subsequently submitted to surgery. A month after VP-shunt implantation these patients showed good clinical improvement and CBF values increased even more. This finding suggests that perfusion MRI (a non-invasive technique) could enhance the selection of candidates for VP shunt implantation.

### Diffusion MRI

In the PP group ADC values decrease significantly in PVWM area due, in our opinion, to the interstitial edema reduction after CSF drainage. However, in BG region a significant increase in ADC values was observed. This phenomenon could be related to relief of the chronic hypoxic environment caused by hydrocephalus. In the NP group no significant variation of ADC values was observed. Correlating clinical and radiological data, the authors found a significant inverse correlation between clinical score and periventricular diffusion values in the PP group in basal and post-test evaluations (i.e. patients with higher diffusion values in PVWM area showed a worse clinical score in the baseline exam). The authors speculated that PVWM diffusion values are related to trans-ependymal interstitial edema; this result is consistent with the findings in literature [19, 22, 33].

### Pathophysiological considerations

Comparing perfusion and diffusion data from basal to post-surgical evaluation, the authors found a significant inverse correlation in PP group. For higher diffusion values, we found lower CBF values. The authors hypothesized that interstitial edema could reduce microvascular blood flow and interfere with the blood supply of these regions. After VP shunt implantation, more CSF was drained; diffusion values decreased (expression of reduced interstitial edema) and perfusion values increased. Draining CSF acutely or chronically decreased the interstitial edema present in PVWM areas in iNPH patients, improving regional blood perfusion and consequently achieving clinical improvement.

When considering the basal ganglia region, the researchers found data that may describe a different pathophysiological mechanism. A significant direct correlation in the PP group between perfusion and diffusion values from baseline to the postsurgical evaluation was demonstrated. In previous studies, a chronic hypoxic environment caused by hydrocephalus has been demonstrated [17, 18, 20]. Researchers considered that the diffusion values calculated in basal ganglia areas could be conditioned mainly by the chronic hypoxic environment causing cytotoxic damage [25, 26, 33], as opposed to what happens in the periventricular areas where the mechanism underlying the chronic damage of the white matter is mainly induced by the interstitial trans-ependymal edema.

After VP shunt implantation, perfusion improved consistently in the basal ganglia; consequently the chronic hypoxic insult decreased as the cytotoxic edema, and diffusion values increase. We postulate that since correlation is significant only when considering variation from the baseline to postsurgical evaluation, a significant amount of time (several weeks) is required to modify the chronic hypoxic environment and improve cytotoxic edema.

Moreover we argued that this different pathophysiological mechanism between periventricular and basal ganglia areas could be explained by the heterogeneity of brain areas and different tissue structure: white matter (with predominance of axons) in PVWM, grey matter (with predominance of cellular bodies) in BG [26].

### Study limitations

There are some underlying limitations of this study. First of all, because of the limited number of patients that influenced the statistical analysis; we were not able to predict some important statistical measures such as positive predictive value of perfusion MRI for selecting patients as candidates for surgery. Another limitation is related to the absence of data that could be derived from negative patients after a hypothetical VP shunt implantation (due to obvious ethical reasons). Those clinical and radiological data could be relevant to confirm the pathophysiological mechanisms we postulate and to point out the role of perfusion and diffusion MRI in the patient selection process.

## Conclusions

This study (despite the limited number of patients) allowed us to observe prospectively diffusion and perfusion changes in probable iNPH patient candidates for a VP shunt. Perfusion MRI (a non-invasive technique) was found to be useful, together with invasive tests, for selecting patients for surgery, increasing the specificity in selected cases. Relationships between diffusion and perfusion values estimated with MRI have been studied and a hypothesis has been proposed to better clarify the pathophysiological mechanisms in iNPH. Further studies conducted on a wider patient population will be required to validate the results observed in this prospective cohort investigation.

## Abbreviations

ADC: apparent diffusion coefficient
BG: basal ganglia
CSF: cerebral spinal fluid
DTI: diffusion tensor imaging
FA: fractional anisotropy
FLAIR: fluid attenuated inversion recovery
ICP: intracranial pressure
iNPH: idiopatic normal pressure hydrocephalus
IR: inversion recovery
MRI: magnetic resonance imaging
MTT: mean transit time
NP: negative patients
PP: positive patients
PVWM: periventricular white matter
PWI: perfusion weighted imaging
rCBF: relative cerebral blood flow
rCMV: relative cerebral blood volume
ROI: region of interest
VP: ventricular-peritoneal
